# Seed Priming Alters the Production and Detoxification of Reactive Oxygen Intermediates in Rice Seedlings Grown under Sub-optimal Temperature and Nutrient Supply

**DOI:** 10.3389/fpls.2016.00439

**Published:** 2016-04-05

**Authors:** Saddam Hussain, Fahad Khan, Weidong Cao, Lishu Wu, Mingjian Geng

**Affiliations:** ^1^College of Resources and Environment, Huazhong Agricultural UniversityWuhan, China; ^2^Institute of Agricultural Resources and Regional Planning, Chinese Academy of Agricultural SciencesBeijing, China

**Keywords:** antioxidant defense system, chilling stress, monoamine oxidase, nutrient deprivation, reactive oxygen intermediates, seed priming

## Abstract

The production and detoxification of reactive oxygen intermediates (ROIs) play an important role in the plant response to nutrient and environmental stresses. The present study demonstrated the behavior of growth, ROIs-production and their detoxification in primed and non-primed rice seedlings under chilling stress (18°C) and nitrogen-(N), phosphorus-(P), or potassium-(K) deprivation. The results revealed that chilling stress as well as deprivation of any mineral nutrient severely hampered the seedling growth of rice, however, seed priming treatments (particularly selenium- or salicylic acid-priming), were effective in enhancing the rice growth under stress conditions. The N-deprivation caused the maximum reduction in shoot growth, while the root growth was only decreased by P- or K-deprivation. Although, N-deprivation enhanced the root length of rice, the root fresh weight was unaffected. Rate of lipid peroxidation as well as the production of ROIs, was generally increased under stress conditions; the K-deprived seedlings recorded significantly lower production of ROIs than N- or P-deprived seedlings. The responses of enzymatic and non-enzymatic antioxidants in rice seedlings to chilling stress were variable with nutrient management regime. All the seed priming were found to trigger or at least maintain the antioxidant defense system of rice seedlings. Notably, the levels of ROIs were significantly reduced by seed priming treatments, which were concomitant with the activities of ROIs-producing enzymes (monoamine oxidase and xanthine oxidase), under all studied conditions. Based on these findings, we put forward the hypothesis that along with role of ROIs-scavenging enzymes, the greater tolerance of primed rice seedlings can also be due to the reduced activity of ROIs-producing enzymes.

## Introduction

Rice, a staple food for more than half of the world’s population, possesses an odd portfolio of tolerance and susceptibility to abiotic stresses compared with other crops. Rice belongs to tropical and subtropical environments and is extremely sensitive to low temperature particularly during early growth stages (Yoshida, 1981). Moreover, rice growth and development is controlled by optimum supply of nutrients like nitrogen (N), phosphorus (P), and potassium (K), and the suboptimal levels of these nutrients is the key limiting factor of rice production throughout the world (Marschner, 1986). Nitrogen is an important component of amino acids and proteins, and it regulates the enzyme activities that are required for energy metabolism including photosynthesis and respiration (Marschner, 1986; De Groot et al., 2003; Epstein and Bloom, 2005). Phosphorus is the constituent of key cellular molecules (such as ATP, nucleic acids, and phospholipids) and has a pivotal role in energy conservation and metabolism (De Groot et al., 2003; Chevalier and Rossignol, 2011; Deng et al., 2014). Likewise, K supply is inevitable for optimum plant growth and development, and it plays important role in plant cellular homeostasis by contributing to osmotic adjustment, charge balance and enzyme catalysis (Marschner, 1986; Epstein and Bloom, 2005; Deng et al., 2014).

The effects of abiotic stresses at whole plant level are usually perceived as a decrease in growth associated with alteration in carbon and N metabolism (Law et al., 2001), and oxidative damage by increased production of reactive oxygen intermediates (ROIs) such as the superoxide radical, hydrogen peroxide, singlet oxygen, and hydroxyl radicals (Mittler, 2002; Gill and Tuteja, 2010). Under optimum growing conditions, plants generally maintain a delicate balance between production and scavenging of ROIs. However, such equilibrium may be perturbed by exposure to stress conditions such as chilling and nutrient deprivation, leading to a sudden increase in intracellular levels of ROIs (Foyer and Noctor, 2003). It has been estimated that 1–2% of oxygen consumption under stress conditions leads to the generation of ROIs in plant tissues (Bhattachrjee, 2005). The production of ROIs occurs from different pathways such as photorespiration, photosynthesis, and mitochondrial respiration. However, it has also been reported that environmental stresses trigger the active production of ROIs by monoamine oxidase (MAO), xanthine oxidase (XOD), and NADPH oxidases (Mittler, 2002). The enhanced ROIs-production under stress negatively affects the cellular and metabolic functions of the plants by damaging the nucleic acids, proteins, lipids, and carbohydrates, and causing lipid peroxidation (Gill and Tuteja, 2010). The perturbation of chloroplastic and mitochondrial metabolism under excessive ROIs-generations also leads to reduced respiration rate and energy supply for growing plant tissue (Taylor et al., 2002).

In order to overcome the excessive ROIs-generation, the plants possess a highly sophisticated and efficient antioxidative defense system. The disruption of cellular homeostasis and stress-induced damages are mitigated by the action of various enzymatic (superoxide dismutase, SOD; catalase, CAT; peroxidase, POD; glutathione peroxidase, GPX; glutathione reductase, GR; glutathione-S-transferase, GST) and non-enzymatic (glutathione, GSH; ascorbic acid or vitamin C, Vc; α-tocopherol or vitamin E, Ve; and carotenoids) antioxidants (Foyer and Noctor, 2005; Gill and Tuteja, 2010; Anjum et al., 2015; Chen et al., 2015). In plants, the levels of ROIs are regulated by the rate of their production/generation and the extent of neutralization by enzymatic and/or non-enzymatic antioxidants. It has been well documented that the activities of antioxidants correlates with the tolerance ability of the plant (Gill and Tuteja, 2010). Chilling-tolerant maize (Hodges et al., 1997; Taka, 2004), cucumber (Kang and Saltveit, 2002), and rice (Huang and Guo, 2005) cultivars had higher antioxidation activities than the sensitive cultivars. Likewise, the enhanced activities of antioxidants protected the plants from oxidative stress caused by the deprivation/deficiency of N (Tewari et al., 2007; Kováčik et al., 2014), P (Tewari et al., 2004, 2007), or K (Tewari et al., 2007; Hafsi et al., 2011).

Seed priming, a controlled hydration technique, has emerged as an effective and indispensable approach to enhance the emergence, seedling vigor and stress tolerance of many field crops including rice (Jisha et al., 2013; Paparella et al., 2015). Plants raised from primed seeds show vigorous start and greater stress tolerance primarily due to more efficient energy metabolism, osmotic adjustment, enlarged embryo, enhanced enzyme activation, and quick cellular defense responses (Jisha et al., 2013). In recent years, seed priming has been proved as a promising approach in modern stress management as it protects plants against pathogens and abiotic stresses without affecting fitness (Chen and Arora, 2013; Jisha et al., 2013). Xu et al. (2011) found that the seed priming improved the chilling tolerance in tobacco during seed germination and seedling growth by the activation of antioxidant system in the plant tissues. Seed priming-induced enhancements in antioxidative defense system of rice seedlings have been well-reported in past (Khaliq et al., 2015; Zheng et al., 2015; Hussain et al., 2016).

In recent years, the generations and scavenging system of ROIs have attracted increasing attention because of their important role in the defense of plants against biotic and abiotic stresses. Although the occurrence of oxidative stress by low temperature (Xu et al., 2011; Hussain et al., 2016), N-deprivation (Kandlbinder et al., 2004; Tewari et al., 2007), P-deprivation (Juszczuk et al., 2001; Malusà et al., 2002; Kandlbinder et al., 2004), and K-deprivation (Shin and Schachtman, 2004; Cakmak, 2005) is well reported in different plant species, the comparative and interactive influence of nutrient deprivation (N, P, or K) and chilling stress on redox and antioxidant status of rice seedlings remains unexplored. Moreover, the role of seed priming on production and detoxification of ROIs under these stress factors is poorly known. Therefore, the present study was performed, for the first time, to characterize the behavior of growth, ROIs-production, and their detoxification in primed and non-primed rice seedlings under chilling stress and N-, P-, or K-deprivation.

## Materials and Methods

### Plant Material

Rice (*Oryza sativa* L.) cultivar “Huanghuazhan” was used in the present experiment. Huanghuazan is an inbred *Indica* cultivar, which is widely cultivated by the rice farmers in central China (Liu et al., 2015). The initial germination and moisture content of the seeds were >95 and <10% (on dry weight basis), respectively. To minimize contamination during priming, seeds were surface sterilized with 2.63% NaOCl solution (household bleach diluted 1:1 with sterile water) for 30 min and rinsed three times with sterile distilled water. Culture tools and hydroponic solution were also sterilized (autoclaved) prior to use.

### Experimentation

The seed-priming treatments were hydropriming (HP; distilled water), chemical priming (Se: 60 μM selenium) and hormonal priming (SA: 100 mg L^-1^ salicylic acid). A non-primed control (NP) was maintained for comparison. The effective levels of these priming solutions were pre-optimized based on rice emergence and early seedling growth performance. Seeds were primed in the dark at 25°C for 24 h with constant gentle agitation. The ratio of seed weight to solution volume (w/v) was 1:5. The priming solution was changed every 12 h (Hussain et al., 2015). After 24 h, the primed seeds were washed with distilled water for 2 min, surface-dried using blotting paper, and transferred to an air-drying oven at 25°C for 48 h to reduce the moisture content to <10%.

The chilling stress (CS) was imposed from the start of experiment in growth chamber by maintaining the day and night temperatures at 18°C, while the temperature (day/night) for control (Cn) treatment was set at 28 C in a separate growth chamber. Under Cn as well as CS conditions, a 12-h light period and humidity of 60% were maintained throughout the study.

Different nutrient management regimes were, [1] sufficient nutrient supply (All Nut), [2] N-deprivation (-N), [3] P-deprivation (-P), and [4] K-deprivation (-K). In All Nut treatments, the nutrient solution, as described by the International Rice Research Institute (Yoshida et al., 1976) was used. The solution for All Nut treatments contained 1 mM (NH_4_)_2_SO_4_, 1 mM NaH_2_PO_4_, 1 mM KCl, 1 mM Ca(NO_3_)_2_, 2 mM Na_2_SiO_3_.9H_2_O, 1 mM MgSO_4_.7H_2_O, 10 μM H_3_BO_3_, 1 μM ZnSO_4_.7H_2_O, 0.5 μM MnSO_4_.H_2_O, 0.1 μM CuSO_4_.5H_2_O, 0.05 μM (NH_4_)6Mo_7_O_24_.4H_2_O, and 20 μM FeNa-EDTA. In treatments of N-, P-, and K-deprivation, all other nutrients were applied at full concentrations except (NH_4_)_2_SO_4_, NaH_2_PO_4_, and KCl, respectively. Different nutrient solutions were imposed at 4 days after sowing (DAS), and all the solutions were renewed after every two days during the course of the study. The pH of solutions was maintained at 6.6 ± 0.2 before supplying to the plants.

**FIGURE 1:**
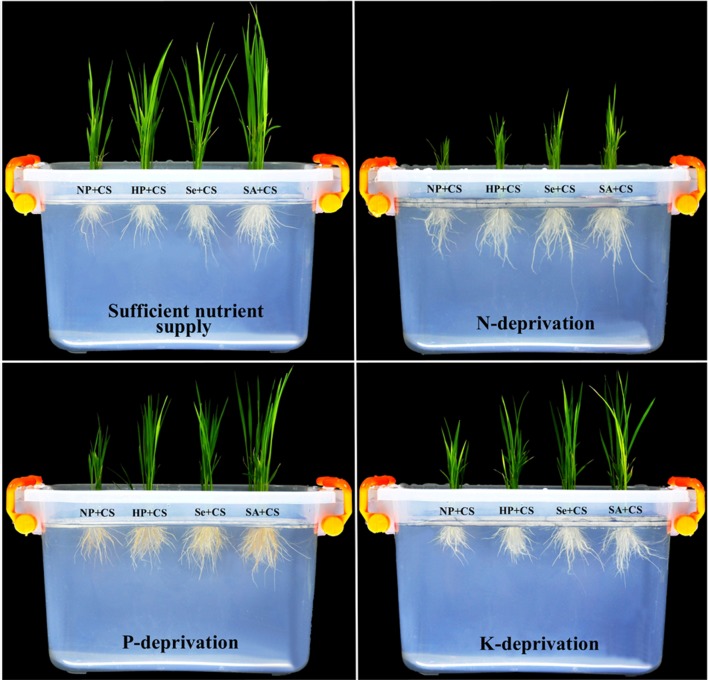
**Pictorial view of primed and non-primed rice seedlings grown under chilling stress and different nutrient management regimes.** Photographs were taken at 15 DAS for treatments under chilling stress (18°C). NP+CS: no priming and 18°C temperature, HP+CS: hydropriming and 18°C temperature, Se+CS: selenium priming and 18°C temperature, SA+CS: salicylic acid priming and 18°C temperature.

Plastic pots (30 cm × 20 cm × 15 cm), containing four liters of respective solution and a floating board on the surface of solution, with four separated sections (for seed priming treatments), were used (**Figure 1**). Twenty seeds of each seed priming treatment were sown in the separated section of board. The experiment was laid out in a completely randomized design with six independent replicates.

### Evaluation of Rice Seedling Growth and Oxidative Metabolism

Seedlings were harvested at 18 DAS, and the photographs (**Figure 1**) were taken 3 days prior to harvesting (15 DAS). Shoot and root length of ten randomly selected seedlings from each replication were measured. Seedlings of each replicate were dissected into roots and shoots, and their fresh weight was recorded immediately using digital electric balance.

All the biochemical analyses were carried out by using fresh leaves samples. Malondialdehyde (MDA) was extracted with 5% trichloroacetic acid (TCA) and was measured using a thiobarbituric acid reaction (Bailly et al., 1996) in order to determine the level of lipid peroxidation. Hydrogen peroxide (H_2_O_2_) content was measured spectrophotometrically at 390 nm (Patterson et al., 1984). The reaction mixture consisted 0.5 ml 0.1% TCA leaf extract supernatant, 0.5 ml of the potassium phosphate buffer (pH 7.0), and 1 ml of 1 M potassium iodide. The amount of H_2_O_2_ in the leaves of rice seedlings was calculated using a standard curve prepared with known concentrations of H_2_O_2_. The MDA as well as H_2_O_2_ content was expressed as μM g^-1^ FW. The contents of hydroxyl ion (OH^-^) and superoxide anion radical ($O_{2}^{•-}$) in the leaves of rice seedlings were determined using the commercial OH^-^ assay kit (A018) and $O_{2}^{•-}$ assay kit (A052), respectively, obtained from Nanjing Jiancheng Bioengineering Institute, China^1^ The OH^-^ was expressed as units mg^-1^ protein, and one unit was the amount required to reduce 1 M of H_2_O_2_ in the reaction mixture per minute at 37°C. The $O_{2}^{•-}$ was demonstrated as units g^-1^ protein, and one unit was equivalent of the value required to inhibit superoxide anion by 1 mg of Vc for 40 min at 37°C.

The activities of ROIs-producing enzymes viz., MAO and XOD were measured by using the kit-A002 and kit-A034, respectively. The activities of both these enzymes were demonstrated as follows: 1 unit mg^-1^ protein for MAO was defined as the amount of enzyme that increased the absorbance by 0.01 at 37°C in 1 hour; 1 unit g^-1^ protein for XOD was defined as 1 g of protein required to transform 1 μM of hypoxanthine to xanthine in 1 min at 37°C.

The activities of enzymatic antioxidants were detected by using the commercial kits in accordance with the manufacturer’s instructions. The kits for SOD (A001), POD (A084-3), CAT (A007-2), GPX (A005), GR (A062), and GST (A004) were purchased from the same company as mentioned above. The absorbance readings of SOD, POD, CAT, GPX, GR, and GST were detected at OD_550_, OD_420_, OD_405_, OD_412_, OD_340_, and OD_412_, respectively (Tecan-infinite M200, Switzerland). The SOD, POD, CAT, GPX, and GST activities were expressed as units mg^-1^ protein, while GR activity was demonstrated as units g^-1^ protein. The units of the antioxidant enzyme activities were defined as follows: one unit of SOD activity was the amount of enzyme required to decrease the reference rate to 50% of maximum inhibition; one unit of POD activity was defined as the amount of enzyme necessary for the decomposition of 1 μg substrate in 1 min at 37°C; one unit of CAT activity was defined as the amount of enzyme required to decompose the 1 μM H_2_O_2_ in 1 second at 37°C; One unit is GPX activity was the amount of enzyme required to oxidize 1 μM GSH in 1 minute at 37°C; one unit of GR activity was defined as the amount of enzyme depleting 1 mM NADPH in 1 min; one unit of GST activity was defined as the amount of enzyme depleting 1 μM GSH in 1 min.

The GSH, Vc, and Ve (kit-A008) content in leaves of rice seedlings were measured using the kit-A006, kit-A009, and kit-A008, respectively. The absorbance for GSH, Vc, and Ve were recorded at OD_420_, OD_536_, and OD_533_ (Tecan-infinite M200, Swit), respectively. The GSH content were expressed as μM g^-1^ protein, the Vc as μg mg^-1^ protein, and Ve as μg g^-1^ tissue fresh weight.

The commercial kit-A015 was used for determination of total antioxidant capability (T-AOC). The absorbance for T-AOC was measured at OD_520_ and data were expressed as unit mg^-1^ protein. One unit of T-AOC was defined as the amount that increased the absorbance by 0.01 at 37°C.

### Statistical Analysis

Data were statistically analyzed following analysis of variance using Statistix 8.1 software (Analytical Software, Tallahassee, FL, USA). Mean variance of the data was analyzed using the Tukey’s HSD (*P* ≤ 0.05) test (Gomez and Gomez, 1984). Comparisons were made and discussed as: (1) among different nutrient management regimes (All Nut, N-deprivation, P-deprivation, and K-deprivation), (2) between non-primed rice seedlings of control temperature (NP+Cn) vs. chilling stress (NP+CS) under different nutrient management regimes, and (3) between NP+CS vs. primed rice seedlings (HP+CS, Se+CS, SA+CS) under different nutrient management regimes.

## Results

### Seedling Growth

**FIGURE 2:**
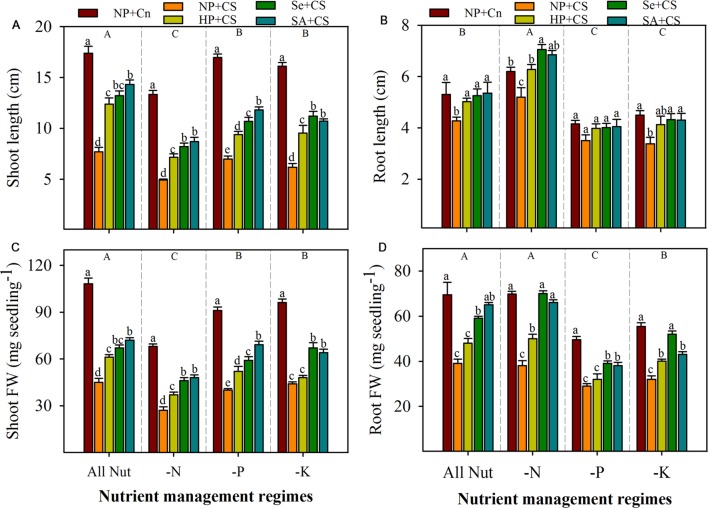
**Shoot length (A), root length (B), shoot fresh weight (C), and root fresh weight (D) of primed and non-primed rice seedlings as influenced by chilling stress and different nutrient management regimes.** Vertical bars above mean indicate standard error of six replicates. Small alphabetical letters (a, b, c…) above means show the differences (*P* ≤ 0.05) among treatments with in a nutrient management regime, while capital alphabetical letters (A, B, C…) reveal the differences (*P* ≤ 0.05) among different nutrient management regimes. NP+Cn: no priming and 28°C temperature, NP+CS: no priming and 18°C temperature, HP+CS: hydropriming and 18°C temperature, Se+CS: selenium priming and 18°C temperature, SA+CS: salicylic acid priming and 18°C temperature, All Nut: sufficient nutrient supply, -N: nitrogen-deprivation, -P: phosphorus-deprivation, -K: potassium-deprivation.

Pronounced variations in the seedling growth of rice were observed under the influence of chilling stress, nutrient deprivation, and seed priming treatments (**Figures 1** and **2**). Irrespective of chilling stress and seed priming treatments, shoot length and shoot fresh weight of rice were significantly reduced after deprivation of N, P, or K, however, such reductions of shoot growth were more for N-deprived seedlings (**Figures 2A,C**). Compared with All Nut, root length was significantly increased, while root fresh weight was statistically similar in N-deprived seedlings, indicating that roots were lengthy but less fibrous under N-deprived conditions. Nevertheless, root length as well as root fresh weight of rice were significantly reduced in P- or K-deprived seedlings (**Figures 2B,D**).

Chilling stress was found to severely reduce the seedling growth of rice under sufficient nutrients supply as well as nutrient deprived conditions. Compared with NP+Cn, root and shoot growth attributes of rice were significantly reduced in NP+CS in all the nutrient management regimes except root length in P-deprivation treatment (**Figures 2A–D**). Chilling-induced reductions in shoot growth of rice were more compared with those in root growth. Likewise, root length was generally less sensitive to chilling stress than root fresh weight. Effects of seed priming were also apparent in alleviating the stress-induced inhibitions of rice seedling growth (**Figures 2A–D**). Compared with NP+CS, all the seed priming treatments significantly increased the shoot length and shoot fresh weight of rice in all the nutrient management regimes except HP+CS for shoot fresh weight under K-deprivation. The Se+CS and SA+CS were generally more effective than HP+CS for enhancing shoot growth of rice (**Figures 2A,C**). These two priming treatments (Se+CS and SA+CS) were also found to significantly increase the root growth attributed of rice compared with NP+CS expect for root length under P-deprived conditions (**Figures 2B,D**).

### Lipid Peroxidation and ROIs-Accumulation

**FIGURE 3:**
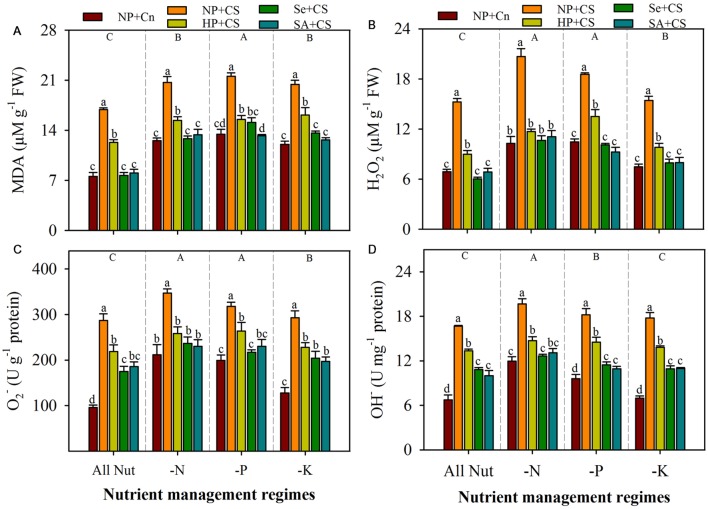
**Lipid peroxidation and accumulation of reactive oxygen intermediates (ROIs) in primed and non-primed rice seedlings under chilling stress and different nutrient management regimes.** MDA content **(A)**, H_2_O_2_ **(B)**, $O_{2}^{•-}$ **(C)**, and OH^-^ **(D)**. Vertical bars above mean indicate standard error of six replicates. Small alphabetical letters (a, b, c…) above means show the differences (*P* ≤ 0.05) among treatments with in a nutrient management regime, while capital alphabetical letters (A, B, C…) reveal the differences (*P* ≤ 0.05) among different nutrient management regimes. Description of treatments is given in **Figure 2**.

Data regarding the rate of lipid peroxidation and accumulation of ROIs in the leaves of primed and non-primed rice seedlings under chilling stress and different nutrient management regimes are shown in **Figure 3**. The lipid peroxidation rates, as indicated by MDA content, were significantly enhanced with deprivation of any mineral nutrient, the maximum for P-deprived seedlings (**Figure 3A**). Likewise, the concentrations of H_2_O_2_ and $O_{2}^{•-}$ were increased to a significant level after nutrient deprivation, particularly for N or P (**Figures 3B,C**). The concentrations of OH^-^ were significantly higher in N- or P-deprived seedlings, but not in K-deprived relative to that in All Nut treatment (**Figure 3D**). Chilling stress triggered the lipid peroxidation and production of ROIs, thus, the accumulations of MDA, H_2_O_2_, $O_{2}^{•-}$, and OH^-^ were significantly higher in NP+CS treatment under all the nutrient management regimes compared with NP+Cn. Seed priming treatments were effective in mitigating the stress-induced enhancement in MDA and ROIs-accumulation. Therefore, the rice seedlings emerged from primed seeds manifested significantly lower MDA contents as well as ROIs-accumulation (**Figures 3A–D**). The Se+CS and SA+CS treatments were generally more effective, and were statistically similar with each other for most of the cases (**Figures 3A–D**).

### Monoamine Oxidase and Xanthine Oxidase Activities

**FIGURE 4:**
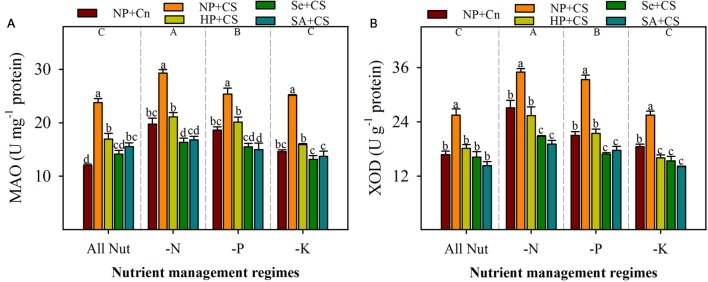
**Activities of monoamine oxidase (MAO) (A) and XOD (B) in primed and non-primed rice seedlings under chilling stress and different nutrient management regimes.** Vertical bars above mean indicate standard error of six replicates. Small alphabetical letters (a, b, c…) above means show the differences (*P* ≤ 0.05) among treatments with in a nutrient management regime, while capital alphabetical letters (A, B, C…) reveal the differences (*P* ≤ 0.05) among different nutrient management regimes. Description of treatments is given in **Figure 2**.

The activities of ROIs-producing enzymes (MAO and XOD) were significantly enhanced with deprivation of N or P, but remained unaffected with K-deprivation (**Figure 4**). The maximum activities of both these enzymes were recorded in N-deprived seedlings. Chilling stress triggered the activities of MAO and XOD, therefore, significantly higher levels of both these enzymes were observed in NP+CS treatments compared with NP+Cn, in all the nutrient management regimes (**Figure 4**). All the seed priming treatments effectively assuaged the stress-induced enhancements in activities of these enzymes, and recorded significantly lower levels of MAO and XOD, compared with NP+CS. Interesting, the activities of both these enzymes in Se+CS and SA+CS treatments were statistically similar or lower than those in NP+Cn, under all the nutrient management regimes except MAO activity in SA+CS for All Nut treatment (**Figure 4**).

### Enzymatic Antioxidants

**FIGURE 5:**
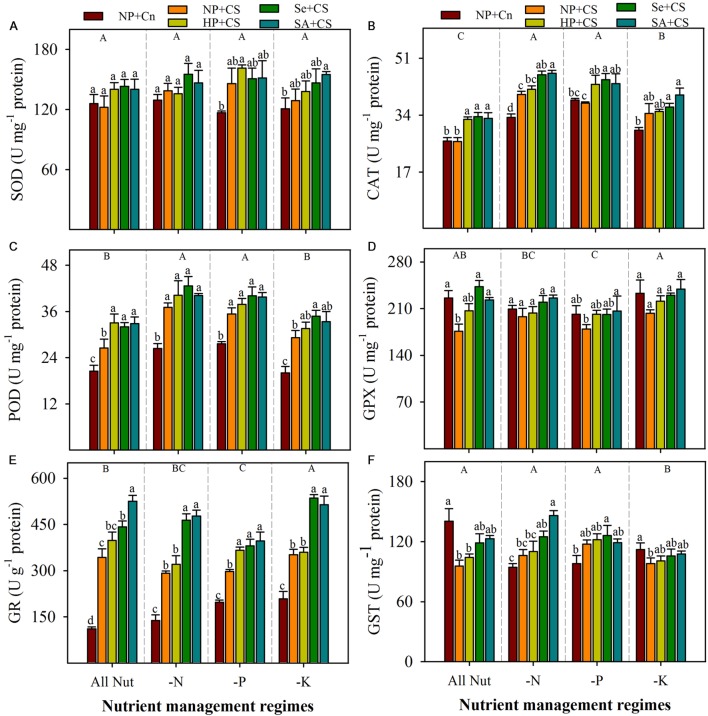
**Activities of enzymatic antioxidants in primed and non-primed rice seedlings under chilling stress and different nutrient management regimes.** SOD **(A)**, CAT **(B)**, POD **(C)**, GPX **(D)**, GR **(E)**, and GST **(F)**. Vertical bars above mean indicate standard error of six replicates. Small alphabetical letters (a, b, c…) above means show the differences (*P* ≤ 0.05) among treatments with in a nutrient management regime, while capital alphabetical letters (A, B, C…) reveal the differences (*P* ≤ 0.05) among different nutrient management regimes. Description of treatments is given in **Figure 2**.

Data regarding antioxidant enzymes activities in the leaves of rice seedlings under the influence of different nutrient management regimes, chilling stress and seed priming treatments are shown in **Figure 5**. The SOD activity in rice seedlings did not alter significantly under deprivation of any nutrient (**Figure 5A**). The CAT and POD activities were significantly increased with N- or P-deprivation, while GPX and GR activities were reduced, particularly with P-deprivation (**Figures 5B–E**). The K-deprived seedlings recorded significantly higher activities of CAT and GR, nevertheless, POD and GPX activities under K-deprivation were statistically similar with All Nut. The GST activity was unaffected by N- or P-deprivation, but reduced with K-deprivation (**Figure 5F**). The effects of chilling stress on antioxidant activities were apparent, but variable with nutrient management regimes. The SOD activity was induced under chilling stress in P- and K-deprived seedlings, but the effects were non-significant. The CAT activities in NP+Cn and NP+CS treatments were statistically similar under All Nut, P-deprivation and K-deprivation, while, NP+CS recorded significantly higher CAT activity under N-deprivation compared with NP+Cn. Interestingly, the POD and GR activities were significantly enhanced after chilling stress in all the nutrient management regimes. Chilling stress decreased the activities of GPX under All Nut treatments, and GST under All Nut and K-deprivation treatments. The GPX as well as GST were unaffected by chilling stress, under N- or P-deprivation (**Figure 5**). The seed priming treatments generally triggered the activities of all antioxidants, but the effects were variable depending on stress conditions. For example, Se+CS and SA+CS significantly increased the activities of CAT, POD, and GPX in All Nut treatments, CAT under N- or P-deprivation, and POD under K-deprivation treatments, compared with NP+CS (**Figure 5**). More apparently, GR activity was significantly enhanced in Se+CS and SA+CS treatments compared with NP+CS under all the nutrient management regimes (**Figure 5E**).

### Non-enzymatic Antioxidants

**FIGURE 6:**
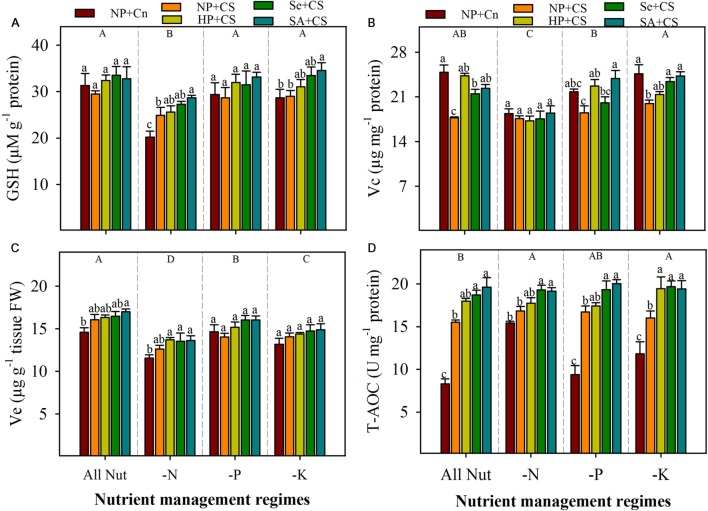
**Non-enzymatic antioxidants and total antioxidant capability of primed and non-primed rice seedlings as influenced by chilling stress and different nutrient management regimes.** GSH **(A)**, Vc **(B)**, Ve **(C)**, and T-AOC **(D)**. Vertical bars above mean indicate standard error of six replicates. Small alphabetical letters (a, b, c…) above means show the differences (*P* ≤ 0.05) among treatments with in a nutrient management regime, while capital alphabetical letters (A, B, C…) reveal the differences (*P* ≤ 0.05) among different nutrient management regimes. Description of treatments is given in **Figure 2**.

The GSH and Vc content in the leaves of rice seedlings were significantly reduced with N-deprivation, but these both antioxidants were unaffected by P- or K-deprivation (**Figures 6A,B**). The Ve content were significantly reduced with the deprivation of any mineral nutrient (**Figure 6C**). Chilling stress did not affect the GSH and Ve content under all the nutrient management regimes, except GSH content were significantly enhanced by chilling stress in N-deprived seedlings. Chilling stress significantly decreased the Vc content in All Nut and K-deprived conditions, but had no affect under N-or P-deprivation. The Ve content were unaffected by seed priming treatments, however, GSH contents were significantly enhanced by SA+CS under N- or K-deprivation. The SA+CS also recorded significantly higher Vc content under All Nut, P-deprived or K-deprived conditions, compared with NP+CS (**Figure 6**).

### Total Antioxidant Capability

The T-AOC in the leaves of rice seedlings was triggered with nutrient deprivation (**Figure 6D**). Compared with All Nut, the N- and K-deprived seedlings recorded significantly higher T-AOC. Chilling stress was found to significantly enhance the T-AOC of rice seedlings under all the nutrient management regimes except N-deprivation, where NP+Cn and NP+CS recorded statistically similar T-AOC (**Figure 6D**). Seed priming regulated the T-AOC in rice seedlings under stress conditions. Compared with NP+CS, the Se+CS and SA+CS recorded significantly higher T-AOC under all the nutrient management regimes (**Figure 6D**).

## Discussion

Under natural conditions, plants usually face with different kinds of abiotic stresses on a daily, seasonal or annual basis. Rice, being a tropical species is extremely sensitive to low temperature particularly at early growth stages. Yoshida (1981) reported that the germination and early growth of rice can be hampered under daily mean temperature of <20°C. In our recent laboratory and field studies, we have found that low temperature (18°C or below) severely hampered the growth of rice seedlings, and disrupted the associated physiological and metabolic processes (Hussain et al., 2016; Wang et al., 2016). In addition to temperature, optimum supply of mineral nutrients (particularly NPK) is also crucial for normal functioning of the plants, and deficiency of any nutrient challenges the survival of plants. Furthermore, to cope with a particular stress conditions (e.g., low temperature), plant require energy and sufficient resources. However, nutrient limitations/deprivation further hinders the acclimation process of plants to stress conditions. As discussed in the introduction, seed priming has been examined and used to enhance of vigor and early growth of many crop plants particularly under stress conditions (Jisha et al., 2013; Khaliq et al., 2015; Zheng et al., 2015; Hussain et al., 2016). The present study investigated, for the first time, the influence of seed priming on growth, ROIs-production, and antioxidant defense system of rice seedlings grown under sub-optimal temperature (18°C) and nutrient supply (N, P, or K-deprivation).

Our results indicated a severe decline in shoot growth attributes of rice with deprivation of any mineral nutrient particularly with N, while root fresh and dry weights were only reduced with the deprivation of P or K (**Figures 1** and **2**). Although the root length was higher in N-deprived seedlings compared with All Nut, the root dry weight was same in these two nutrient management regimes (**Figures 2B,D**) suggesting that the roots of N-deprived seedlings were less fibrous and thin. Similar responses were also observed in *Arabidopsis thaliana*, even after 2-days of N starvation (Scheible et al., 2004). In the present study, the shoots of N-deprived seedlings were also less greenish showing the N deficiency. There were no apparent deficiency symptoms in the shoots of P- or K- deprived seedlings, although a change in root appearance was evident (**Figure 1**). In P-deprived seedlings, more lateral roots and shorter primary roots were observed and the color of roots was brownish, which might be due to high iron accumulation (**Figure 1**). Although the iron concentration was not measured in the present study, the high accumulation of iron under P deficiency is well-reported in the past (Zheng et al., 2009).

Chilling stress severely hampered the shoot and root growth of rice seedlings, which might be attributed to reduced cell division and elongation, low respiration rate, and high oxidative stress under low temperature, as observed previously (Hussain et al., 2016; Wang et al., 2016). Seed priming treatments were effective in alleviating the stress-induced adversities on seedling growth of rice; the Se- and SA-priming treatments were more effective than hydropriming (**Figures 1** and **2**). The beneficial effects of these priming reagents has been reported in the past under different stress conditions (Gunes et al., 2007; Jisha et al., 2013; Hussain et al., 2016; Wang et al., 2016).

At a whole plant level, abiotic stresses lead to the formation of excessive ROIs that can generate oxidative stress by oxidizing lipid, nucleic acids, and proteins (Mittler, 2002; Gill and Tuteja, 2010). Accordingly in the present study, chilling and nutrient deprivation triggered the production of H_2_O_2_, $O_{2}^{•-}$, and OH^-^ in the rice seedlings (**Figures 3B–D**). The enhanced levels of these ROIs were also coincided with the higher lipid peroxidation rate (**Figure 3A**), which is regarded as a biochemical marker for the free radical mediated injury in plants. These results are in line with previous researchers, who observed enhanced ROIs-production and MDA content under chilling stress (Ruelland et al., 2009; Xu et al., 2011; Hussain et al., 2016), N-deprivation (Kandlbinder et al., 2004; Tewari et al., 2004, 2007), P-deprivation (Juszczuk et al., 2001; Malusà et al., 2002; Kandlbinder et al., 2004), and K- deprivation (Cakmak, 1994; Shin and Schachtman, 2004; Tewari et al., 2004, 2007). Compared with N- or P-deprivation, lower accumulation of ROIs was observed in K-deprived seedlings (**Figure 3**), which corroborate with earlier observations on K-deficient maize (Tewari et al., 2004) and mulberry (Tewari et al., 2007). All the seed priming treatments recorded significantly lower accumulation of ROIs and lipid peroxidation compared with NP+CS (**Figure 3**) which indicates that oxidative stress (caused by low temperature and nutrient deprivation) and seedling damage were effectively assuaged after seed priming. Previously in rice, similar beneficial effects of seed priming have also been observed under drought (Zheng et al., 2015) and chilling stress (Hussain et al., 2016).

Generally, photorespiration, photosynthetic apparatus, and mitochondrial respiration are considered as the main sources for the generation of ROIs under normal conditions. However, some other sources such as MAO and XOD, also contribute in the active production of ROIs particularly under environmental stresses (Mittler, 2002). The MAO, a flavoprotein localized on the outer membrane of mitochondria, catalyzes the oxidative deamination of aromatic amines and produces a large quantity of H_2_O_2_, which ultimately contributes to an increase in the steady state concentrations of ROIs within the plant cell (Cadenas and Davies, 2000). Likewise, XOD can generate the toxic $O_{2}^{•-}$ as well as H_2_O_2_ in the plant cell (Schrader and Fahimi, 2006), and sometimes, high production of $O_{2}^{•-}$ from a XOD system cause epidermal cell death, that cannot be prevented by plant defense system. In the present study, MAO and XOD were considerably altered under the influence of chilling stress and nutrient deprivation; the activities of both these enzymes were concomitant with the levels of ROIs in the rice seedlings (**Figures 3** and **4**). For instance, significantly higher levels of MAO and XOD under N- or P-deprivation, led to the greater production of ROIs under these conditions. While, comparative lower levels of MAO and XOD in K-deprived seedlings were consistent with lower accumulation of ROIs (**Figures 3** and **4**). It was found that all the seed priming treatments significantly reduced the activities of MAO and XOD (**Figure 4**), which indicate a possible reason of reduced ROIs-accumulation and oxidative stress in primed rice seedlings.

The antioxidant defense system of plants mainly including enzymatic (SOD, POD, CAT, GPX, GR, GST etc.) and non-enzymatic (GSH, Vc, Ve etc.) antioxidants, is regarded to overcome the cascades of uncontrolled oxidation and protect the plant cells from ROIs-induced oxidative damage (Foyer and Noctor, 2005; Gill and Tuteja, 2010; Anjum et al., 2015; Chen et al., 2015). The SOD plays a key part in catalyzing the dismutation of $O_{2}^{•-}$, while CAT, POD, and GPX contribute in scavenging of H_2_O_2_ (Gill and Tuteja, 2010; Anjum et al., 2015; Fahad et al., 2015). In present study, CAT and POD were significantly enhanced with N- or P-deprivation, and their activities were well related with increased H_2_O_2_ concentration in these treatments (**Figures 3** and **5**). Accordingly, comparatively lower activities of these enzymes in K-deprived seedlings were concomitant with the less H_2_O_2_ generation (**Figures 3** and **5**). Increase in CAT and POD activities in N- or P-deprived rice seedlings is in agreement with previous studies on N or P-deprived mulberry (Tewari et al., 2007), N-deprived wheat (Polesskaya et al., 2004), P-deficient maize (Tewari et al., 2004), and P-deficient bean (Juszczuk et al., 2001) plants. Compared with other H_2_O_2_-scavenging enzymes, POD was highly triggered under chilling stress (**Figure 5C**), which indicates that this enzyme was more active under stress conditions. He et al. (2010) also reported that POD was more responsive to chilling stress than other antioxidant enzymes. In the present study, activities of GR and GST were also influenced by chilling stress and different nutrient management regimes but their responses were variable with stress conditions (**Figures 5E,F**). The GR activity was significantly enhanced with K-deprivation as well as chilling stress, while reduced with P-deprivation. Tewari et al. (2007) also found that K-deprivation enhanced the GR activity in mulberry leaves, while P-deprived plants recorded the lower GR activity than those with N- or K-deprivation. Moreover, high GR activities in rice under chilling stress have also been demonstrated by Huang and Guo (2005).

Non-enzymatic antioxidants like GSH, Vc, and Ve also play key role in stress tolerance of crop plants. The GSH and Vc are involved in many cellular processes under stress, and can directly detoxify ROIs and thus contribute to non-enzymatic ROIs-scavenging (Foyer and Noctor, 2005; Gill and Tuteja, 2010). The Ve is regarded to maintain the membrane stability, by quenching or scavenging ROIs like singlet oxygen. In the present study, the GSH, Vc, and Ve were reduced with N-deprivation (**Figure 6**), which was consistent with high ROIs-production and poor growth performance in these treatments (**Figures 1–3**). Likewise, higher GSH and Vc activities were correlated with lower ROIs-production, in K-deprived seedling. Chilling stress did not affect Ve, but altered the GSH and Vc contents depending on the nutrient management regimes. Total antioxidant capability of rice seedlings was generally, regulated with nutrient deprivation as well as chilling stress (**Figure 6D**), which clearly indicate the occurrence of oxidative stress. Considering all the antioxidants, it was clear that although the antioxidants were generally triggered under stress, yet their levels were not enough to overcome the increasing level of ROIs. The disproportionate increase of ROIs-producing oxidases (e.g., MAO, XOD), in comparison to ROIs-scavenging enzymes (**Figures 4–6**) might be suggested to be responsible for oxidative stress, which hampered the seedling growth under sub optimal temperature and nutrient supply.

All the seed priming treatments were found to trigger or at least maintain the antioxidant defense system of rice seedlings compared with NP+CS under all the nutrient management regimes (**Figures 5** and **6**). The positive role of seed priming in regulating enzymatic and non-enzymatic antioxidants has been well established under different kinds of abiotic stresses (Xu et al., 2011; Zheng et al., 2015; Hussain et al., 2016). In the present study, GR and T-AOC were significantly enhanced by Se+CS and SA+CS compared with NP+CS; the positive effects of seed priming treatments on the other enzymatic and non-enzymatic antioxidants were rarely significant (**Figures 5** and **6**). Interestingly, the accumulations of ROIs were significantly lower in all the seed priming treatments (**Figure 3**), indicating that the reduced activities of ROIs-producing enzymes (MAO and XOD activities) in primed rice seedlings (**Figure 4**) might be the possible reason for such clear difference along with the role of scavenging antioxidants. Chen and Arora (2013) proposed that seedling emerged from primed seeds cope with environmental stresses by vigorous head-start or/and cross tolerance. During priming process, early imbibition process promotes the efficient mitochondrial development by augmenting energy metabolism, while after rehydration of primed seeds, main cellular processes such as the *de-novo* synthesis of nucleic acids and proteins, ATP production, activation of DNA repair and antioxidant mechanisms are triggered leading to higher stress tolerance ability. Several studies on different abiotic stresses have confirmed that enhanced antioxidant levels during priming helped rice seedlings to overcome the stress-induced challenges after germination, such as ROIs-production (Bailly et al., 2000, 2008; Khaliq et al., 2015; Zheng et al., 2015). In the present study, 18-days-old seedlings were analyzed for the measurements of ROIs and antioxidants, in order to examine the effects of temperature and nutrient treatments. It can be assumed that reduced ROIs-production and activities of MAO and XAO in primed rice seedlings, might also be the effect of strong anti-oxidative defense system during early phase of seedling growth. This assumption is also supported by our previous study on chilling stress (Hussain et al., 2016), where we found that effects of seed priming were more prominent on antioxidant activities of 10-days old rice seedlings. Due to slow growth rate of rice seedlings under chilling stress, we have to extend the harvesting time for at least 18 DAS in the present study, in order to ensure that all the nutrients from the grains were exhausted and the conditions were nutrient-deprived. It would be interesting to observe the response of ROIs-production and detoxification in primed and non-primed rice seedlings at different periods of time under multiple stress factors. Moreover, investigating the molecular mechanisms of priming-induced effects at transcriptomic and proteomic levels will further strengthen the concepts.

## Conclusion

Chilling stress as well as deprivation of N, P, or K severely hampered the seedling growth of rice, however, seed priming treatments, particularly Se- and SA-priming, were effective in enhancing the rice growth under stress conditions. The N-deprivation caused the maximum reduction in shoot growth, while root growth was only decreased by P- or K-deprivation. The N-deprivation enhanced the root length of rice, nevertheless, root fresh weight was unaffected. Rate of lipid peroxidation and as well as the production of ROIs was generally increased under stress conditions; the K-deprived seedlings recorded significantly lower production of ROIs compared with those under N- or P-deprived conditions. The responses of enzymatic and non-enzymatic antioxidants in rice seedlings were variable with stress condition. All the seed priming were found to trigger or at least maintain the antioxidant defense system of rice seedlings. More interestingly, the levels of ROIs were significantly reduced by seed priming treatments, which were consistent with the activities of MAO and XOD, under all studied conditions. These findings suggested that the reduced activity of ROIs-producing enzymes was also the main reason for better tolerance of primed rice seedlings, along with the role of ROIs-scavenging enzymes.

## Author Contributions

SH and MG initiated and designed the research, SH and FK performed the experiments and collected the data, SH, FK, and MG analyzed the data and wrote the manuscript. WC and LW edited the manuscript and provided guidance during experimentation.

## Conflict of Interest Statement

The authors declare that the research was conducted in the absence of any commercial or financial relationships that could be construed as a potential conflict of interest.

## Footnotes

1 www.njjcbio.com
